# Perphenazine Attenuates the Pro-Inflammatory Responses in Mouse Models of Th2-Type Allergic Dermatitis

**DOI:** 10.3390/ijms21093241

**Published:** 2020-05-03

**Authors:** Min-Jeong Heo, Soo Young Choi, Chanmi Lee, Yeong Min Choi, In-sook An, Seunghee Bae, Sungkwan An, Jin Hyuk Jung

**Affiliations:** 1 Korea Institute of Dermatological Science, GeneCellPharm Corporation, 375 Munjeong 2(i)-dong, Songpa-gu, Seoul 05836, Korea; alselddlrh@skinresearch.or.kr (M.-J.H.); Choisy@skinresearch.or.kr (S.Y.C.); chanmi2026@skinresearch.or.kr (C.L.); sharmine@skinresearch.or.kr (Y.M.C.); anis@skinresearch.or.kr (I.-s.A.); 2 Research Institute for Molecular-Targeted Drugs, Department of Cosmetics Engineering, Konkuk University, Seoul 05029, Korea; sbae@konkuk.ac.kr

**Keywords:** dopamine receptor D2, perphenazine, dermatitis, drug repurposing

## Abstract

Developing dermatitis therapeutics has been faced with challenges including adverse effects of topical steroid and high cost of new developing drugs. Here, we found the expression levels of dopamine receptor D2 is higher in skin biopsies of dermatitis patients and an oxazolone-induced animal model of dermatitis. We used perphenazine, an FDA-approved dopamine receptor antagonist to determine the therapeutic effect. Two different animal models including 12-o-tetradecanoylphorbol-13-acetate (TPA) and oxazolone (OXA)-induced dermatitis were employed. TPA and OXA-mediated ear swelling was attenuated by perphenazine. Moreover, perphenazine inhibited infiltrated mast cells into lesion area. We found levels of serum IgE, histamine and cytokines are decreased in mice cotreated with perphenazine and OXA compared to OXA-treated mice. Overall, this is a first study showing that the FDA-approved, anti-psychotic drug, perphenazine, alleviates animal models of dermatitis.

## 1. Introduction

Dermatitis is a skin inflammatory condition which occurs in many forms including spongiosis, dry red skin, rash and swollen [1]. Atopic dermatitis (AD) is one of the frequent types of dermatitis that occurs in children [2]. Topical steroids are one of the major dermatitis therapeutics but its adverse effects have been well established [3,4]. Non-steroid drugs, including Type 4 phosphodiesterase (PDE4) inhibitors as well as JAK-STAT inhibitors, have been developed for dermatitis therapeutics [5,6]. Those developing drugs are expected to have less adverse effects than steroids because of their specificity on the molecular target [5]. In addition, abnormal Th2 immune responses are frequently found in AD and it is considered to be one of the potent targets for therapeutic development t [7,8]. Especially, antibodies against IL-4 and IL13 have been investigated as molecular targets for moderate-to-severe atopic dermatitis [9,10]. Dopamine has been widely recognized as a neurotransmitter of the brain as well as the peripheral system [11]. Recent studies support that dopamine plays an important role in the immune system [12,13]. Dopamine receptors D1 and D2 are expressed in many immune cells and the dopamine receptor antagonist ameliorates inflammatory diseases [14,15,16,17]. Moreover, dopamine receptor D3 signaling in CD4 T cells favoring Th1 and Th17 mediated responses in disorder involving reduction of dopamine levels in target tissue, such as Parkinson’s diseases and inflammatory bowel diseases [18,19]. However, dopamine signaling in dermatitis is poorly understood although various immune responses are involved in pathophysiological event in dermatitis [20]. Dopamine antagonists block dopamine receptor signals [21]. Especially, Perphenazine is a dopamine receptor D1 and D2 antagonist that is considered an antipsychotic [22]. Here, we determine dopamine receptor expression in dermatitis and effect of its antagonist in animal models of dermatitis.

## 2. Results

### 2.1. Dopamine Receptor D2 is Highly Expressed in Dermatitis Patients and Animal Model of Dermatitis

**Figure 1:**
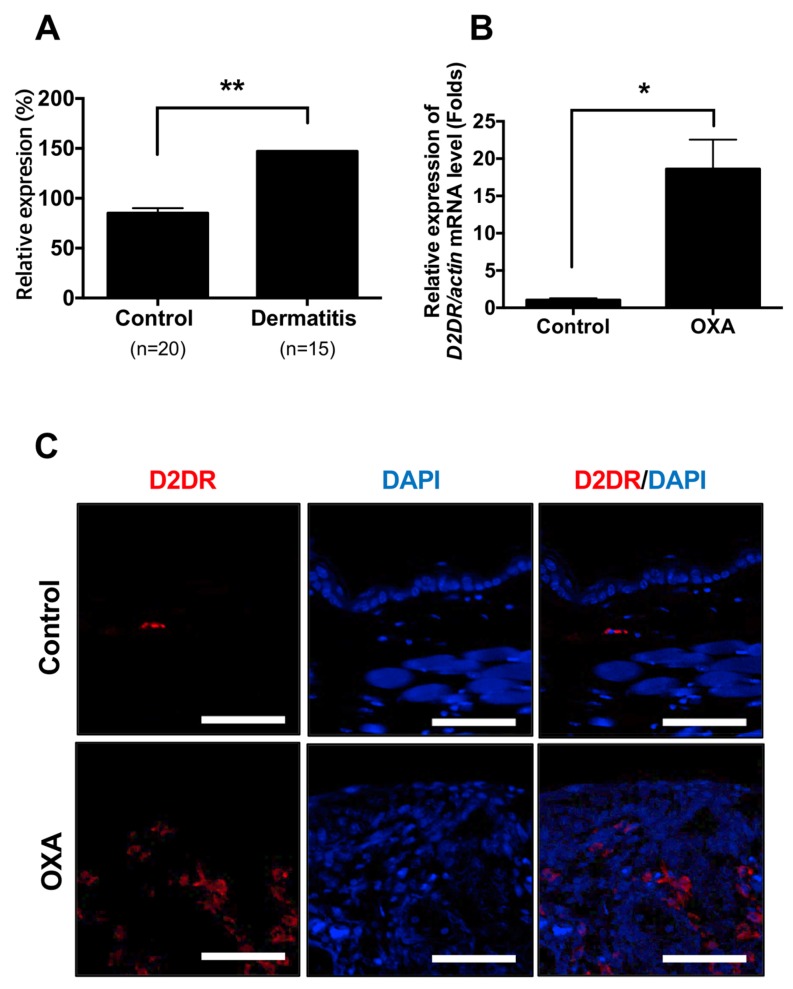
The expression levels of dopamine receptor D2 are increased from dermatitis patients and animal model of dermatitis (**A**) Expression levels of dopamine receptor D2 in normal (*n* = 20) and dermatitis patients (*n* = 15). (**B**) mRNA levels of dopamine receptor D2 (D2DR) from OXA-treated mice. Data are presented as mean ± SEM and analyzed by student *t*-test. * *p* < 0.05 and ** *p* < 0.01 vs. control. (**C**) Immunofluorescent analysis of dopamine receptor D2 (D2DR) from control and oxazolone-treated mice ear. Scale bar = 60 µm.

While we were screening a molecular target for dermatitis using meta-analysis, we found the expression levels of dopamine receptor D2 were increased from datasets of atopic dermatitis patients (GSE6012 [23,24], and GSE120721 [25] (Figure 1A and Table S1). Consequently, dopamine receptor D2 was increased from the ear of OXA-treated mice, especially in the dermis where many immune cells are infiltrated (Figure 1B,C). These results indicated the levels of dopamine receptor D2 are increased in atopic dermatitis patients as well as in animal model of dermatitis.

### 2.2. Perphenazine Ameliorates TPA-Induced Animal Model of Dermatitis

**Figure 2:**
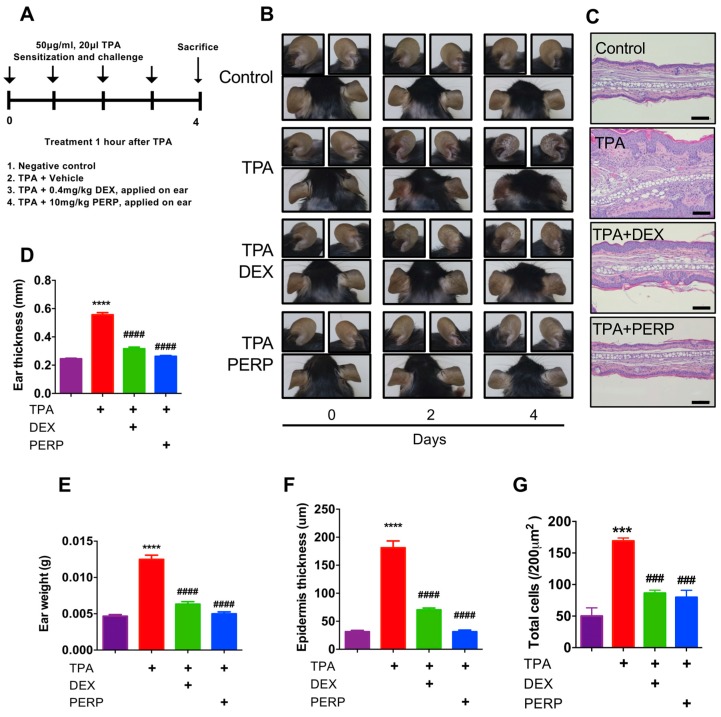
Perphenazine attenuates TPA-induced acute dermatitis model in mice. (**A**) Schematic diagram of TPA-induced animal model. Four groups: untreated controls, TPA only and mice treated with DEX (Dexamethasone) or Perphenazine (PERP) one hour after every TPA challenge (*n* = 7). (**B**) Representative pictures of mouse ears collected at zero, two, and four days. (**C**) Histological sections of ear biopsies were analyzed using H&E-stained sections. Original magnification = X200. Scale bar = 100 um. (**D**) Ear thickness and (**E**) Ear weight. (**F**) Epidermal thickness of the ear skin. (**G**) Total infiltrated cells were counted. Data are presented as mean  ±  SEM and analyzed by one-way ANOVA (*** *p* < 0.005 and **** *p* < 0.001 vs. control) and (### *p* < 0.005 and #### *p* < 0.001 vs. TPA).

Peripheral dopamine has been previously reported as an immune modulator, which serves an important role in immune cell regulation. However, a functional aspect of peripheral dopamine receptor D2 has been poorly understood. Therefore, we used perphenazine, a dopamine receptor antagonist to determine whether perphenazine attenuates animal models of dermatitis. 12-o-tetradecanoylphorbol-13-acetate (TPA) induces skin inflammation, infiltrated immune cells and epidermal hyperplasia [26,27]. We employed a TPA-induced animal model to determine whether perphenazine is effective on dermatitis (Figure 2A). Four days of perphenazine treatment was able to attenuate inflammatory phenotypes including skin redness (Figure 2B). Consequently, ear thickness and weight were decreased from mice cotreated with perphenazine and TPA compared to TPA-treated mice (Figure 2D,E). We found that TPA-induced skin edema as well as epidermis thickness are significantly decreased in mice cotreated with perphenazine and TPA compared to TPA-treated mice using histological analysis (Figure 2C,F). Total infiltrated cells were decreased in mice cotreated with perphenazine and TPA compared with TPA-treated mice (Figure 2G). These results indicated that perphenazine is effective on an animal model of acute dermatitis.

### 2.3. Perphenazine Ameliorates Morphological Phenotype of Oxazolone-Treated Animal Model of Dermatitis

**Figure 3:**
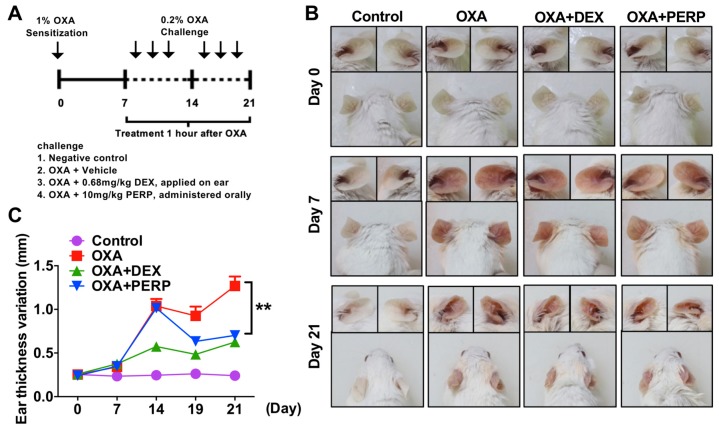
Perphenazine alleviates morphological phenotype of OXA-induced dermatitis model in mice. (**A**) Schematic diagram of an OXA (Oxazolone)-induced animal model. Four groups: untreated controls, OXA only and mice treated with DEX (Dexamethasone) or Perphenazine (PERP) one hour after every OXA challenge (*n* = 7). (**B**) Representative photographs of mouse ears from each group on day zero, seven, and 21. (**C**) Ear thickness was measured every week as indicated. Data are presented as mean ± SEM and analyzed by the student t-test. ** *p* < 0.01 vs. oxazolone treated.

As perphenazine is effective on attenuation of TPA-induced dermatitis, we further investigated whether perphenazine alleviates atopic dermatitis in an animal model. Oxazolone is widely used in the atopic dermatitis animal model [28]. The redness of the mice ears was attenuated in mice cotreated with oxazolone and perphenazine compared to OXA-treated (Figure 3A,B). The levels of mice ear swelling were also decreased in mice cotreated with OXA and perphenazine compared to oxazolone-treated (Figure 3C).

### 2.4. Perphenazine Ameliorates Histological Phenotype of Oxazolone-Treated Mice

**Figure 4:**
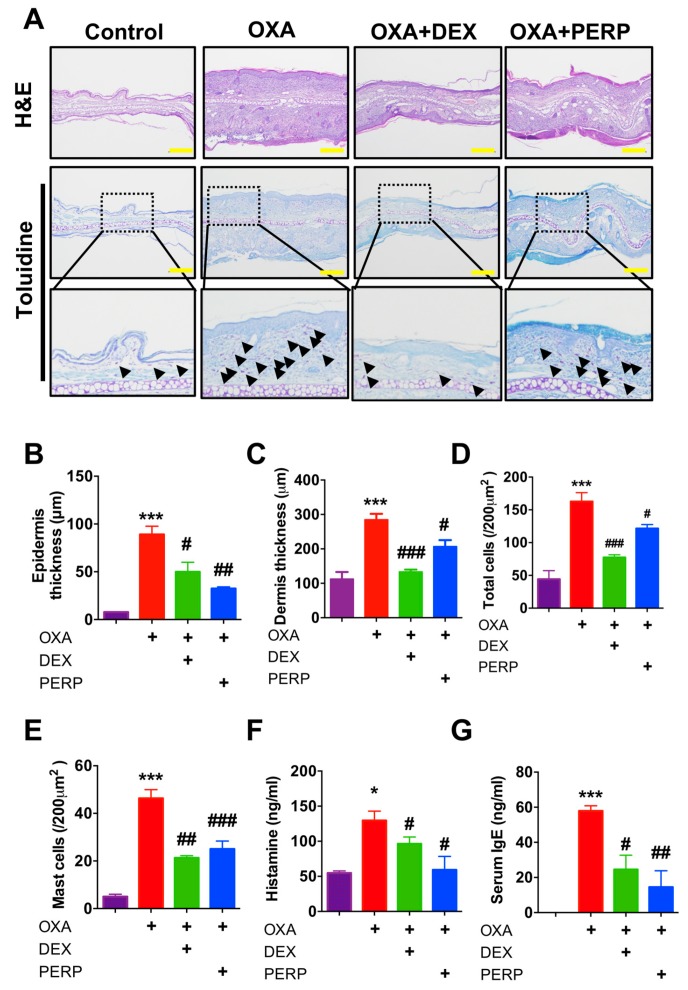
Perphenazine alleviates histological phenotype of OXA-induced dermatitis model in mice. (**A**) H&E staining and toluidine blue staining in ear lesions (**B**) Epidermal thickness (μm) was determined by micrometer. (**C**) Dermis thickness (μm) was determined by micrometer. (**D**) Total cells were counted. (**E**) Mast cells (black arrow) in dermis were counted. (**F**) Serum histamine levels were measured (**G**) Serum IgE levels were measured. Scale bar, 100μm. Data are presented as mean ± SEM of changes in values and analyzed by one-way ANOVA. (* *p* < 0.05, ***p < 0.005 compared to control) and (# *p* < 0.05, ## *p* < 0.01, and ### *p* < 0.005 vs. oxazolone treated).

Consequently, we found levels of epidermis and dermis thickness are decreased in mice cotreated with OXA and perphenazine compared to OXA-treated (Figure 4A–C). Moreover, numbers of infiltrated total cell as well as mast cells were also decreased in mice cotreated with OXA and perphenazine compared to OXA-treated (Figure 4D,E). Moreover, serum histamine as well as IgE levels were decreased in mice cotreated with OXA and perphenazine compared to OXA treated mice (Figure 4F,G). These results indicated perphenazine ameliorates oxazolone-induced dermatitis in a mice model.

### 2.5. Perphenazine Attenuates Cytokine Expression in Oxazolone-Treated Mice

**Figure 5:**
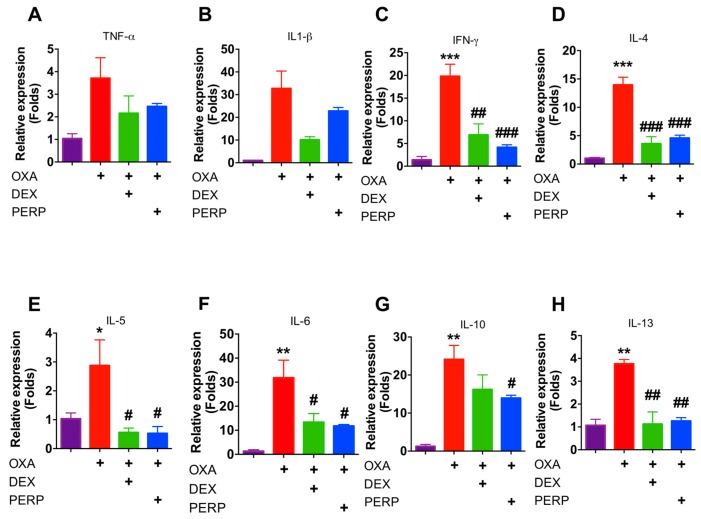
Perphenazine reduces the expression levels of cytokine in the lesion of OXA-induced dermatitis in mice mRNA expression of (**A**) TNF-α (**B**) IL-1β (**C**) IFN-χ (**D**) IL-4 (**E**) IL-5 (**F**) IL-6 (**G**) IL-10 (**H**) IL-13 were determined from indicated mice ear. Data are presented as mean ± SEM and analyzed by one-way ANOVA (* *p* < 0.05, ** *p* < 0.01, *** *p* < 0.005 vs. control) and (# *p* < 0.05, ## *p* < 0.01, ### *p* < 0.005 vs. oxazolone treated).

As Th2 cytokines including IL-4 and IL-13 are pathological markers of atopic dermatitis, we analyzed the levels of these cytokines to determine whether perphenazine attenuates cytokine expression [29]. Interestingly, levels of Th1 and Th2 cytokines were decreased from mice cotreated with perphenazine and OXA compared to OXA-treated mice (Figure 5A–H). These results indicated that perphenazine attenuates cytokine expression in oxazolone-induced mice. As NFκB signaling is one of the primary modulators in inflammation as well as cytokine expression, we determined whether perphenazine attenuates NFκB activation using 3T3 cells, which expressed luciferase reporter plasmid. As 0.1-nM perphenazine-treated fibroblasts showed 87% viability, 0.01- and 0.1-nM perphenazine were used to determine NFκB activity (Figure S2A). We found that perphenazine is not able to regulate NFκB activity (Figure S2B).

## 3. Discussion

We found the expression levels of dopamine receptor D2 are relatively higher in the lesion of dermatitis patients compared to control subjects and future study remains to determine which type of immune cells are responsible for the expression of dopamine receptor in the dermatitis lesion (Figure 1C and Table S1). Recent studies support that peripheral dopamine receptor (DRD1 and DRD3) are expressed in T cells and dendritic cells [30,31,32]. Thus, infiltrated T cells into the dermatitis lesion may express dopamine receptor D2. Perphenazine is an anti-psychotic FDA-approved drug for schizophrenia [22]. We used dexamethasone, a widely used steroid to compare therapeutic efficacy with perphenazine. Interestingly, perphenazine treatment alleviates OXA-induced dermatitis significantly (Figure 3B,C). Moreover, perphenazine treatment showed better therapeutic effect than dexamethasone on the decrease of epidermal thickness from two different animal models (Figure 2E and Figure 4B). We measured mice weight because perphenazine has sedative effects and leads to the loss of animal weight. Mice weight loss was not observed in perphenazine treated mice (Figure S1). In fact, we used a relatively low amount of perphenazine compared to a recent study showing the anti-cancer activity of perphenazine [33]. We found that perphenazine reduces levels of cytokine expression including IL-4 and IL-13 (Figure 5A–H). As NFkB signaling is one of the primary modulators in inflammation, we determined whether perphenazine attenuates NFκB activation (Figure S2B). We found that perphenazine is not able to regulate NFkB in vitro. Consequently, we focused on mast cell regulation by perphenazine because infiltrated mast cells as well as serum histamine levels were decreased in mice cotreated with perphenazine and OXA (Figure 4D,E). Our data correlate with recent reports that dopaminergic drugs inhibit mast cell granulation and immune response [34]. In addition, perphenazine is a phenothiazine derivative. Among phenothiazines, there is promethazine, a potent antihistamine [35]. Therefore, the anti-inflammatory effect of perphenazine may be related with itch response by inhibition of mast cell infiltration and histamine release. Future study of Drd2-positive cells in AD pathogenesis remains to elucidate the peripheral dopamine signaling in itch response. On the other hand, perphenazine showed anti-cancer activity by blocking cholesterol metabolism [33]. As the metabolic shift of immune cells is essential for activation, it would be interesting to determine whether perphenazine modulates immune cell metabolism in dermatitis. In conclusion, we found elevation of dopamine receptor expression in skin biopsies of dermatitis patients and an animal model of dermatitis. The dopamine receptor antagonist perphenazine, alleviates animal models of dermatitis and further studies are required to investigate molecular mechanism of perphenazine-mediated effect on dermatitis to expand the potential of perphenazine as a repurposing drug for dermatitis.

## 4. Materials and Methods

### 4.1. Experimental Animals

For oxazolone-induced animal model, seven-week-old BALB/c mice were purchased from the Central Laboratory Animals (Seoul, Korea) and used after one week for quarantine. For the TPA-induced animal model, seven-week-old C57/B6 mice were purchased from Nara Biotech (Seoul, Korea) and used after one week for quarantine. Mice were housed in the animal cage under environment condition as temperature (20 ± 2 °C) / humidity (50 ± 5%) and maintained under specific pathogen-free conditions with a 12-h light/dark cycle. All experimental procedures were approved by the Institutional Animal Care and Use Committee of the Konkuk University (KU19160, 4 September 2019).

### 4.2. TPA-Induced Acute Dermatitis in Mice

TPA-induced acute dermatitis was induced in the mice (*n* = 7) by topical application of 12-o-tetradecanoylphorbol-13-acetate (TPA (Sigma-Aldrich, St. Louis, MO, USA)) as previously described [26]. Dexamethasone (Sigma-Aldrich, St. Louis, MO, USA) (0.4 mg/kg) was topically administrated to the ears of mice or perphenazine (10 mg/kg) (PERP; TCI America, Tokyo, Japan), and was orally administrated after 1 h of TPA treatment. Concentration of perphenazine was selected according to previous reports [35,36]. For skin inflammation signs, ear thickness was measured prior to each TPA application using a digital caliper (Mitutoyo, Tokyo, Japan) on days zero, two and four to evaluate ear swelling reactions. No substances were administrated to the ear surfaces on the last day of the experiment. Experimental measurements were performed by the same trained investigator. After four consecutive days, mice were sacrificed and 5-mm diameter ear biopsies were obtained with a punch (Kai Industries, Gifu, Japan). Ear biopsies were weighed and collected for histopathological analysis. All experimental procedures were approved by the Institutional Animal Care and Use Committee of the Konkuk University (KU19160).

### 4.3. OXA-Induced Murine Model of Dermatitis

The oxazolone-induced animal model was prepared as previously described [26,37]. The mice were divided into four groups (*n* = 7). Negative control was sensitized and challenged with phosphate-buffered saline (PBS). OXA group was treated with oxazolone (4-Ethoxymethylene-2-phenyl-2-oxazolin-5-one; Sigma-Aldrich, St. Louis, MO, USA). For therapeutic groups, 0.68 mg/kg of dexamethasone (DEX) was applied on ear and 10 mg/kg of perphenazine (PERP; TCI America, Tokyo, Japan) was administered orally 1 h after OXA challenge. Mice were photographed by Digital single-lens reflex camera (F5.6 1/40, ISO800; Canon, Tokyo, Japan) on days zero, seven, and 21. Ear tissues were used for staining of H&E and toluidine blue. All experimental procedures were approved by the Institutional Animal Care and Use Committee of the Konkuk University (KU19160).

### 4.4. Histology

Histological analysis was performed as previously described [26]. Briefly, the ear tissues were collected using 5-mm biopsy punches (KAI Medical, Japan) and fixed in 10% formaldehyde solution. Tissues were processed using standard methods (from 70% to 100% ethanol and xylene step) for H&E and toluidine blue staining. The stained tissues were observed at 200× magnification under a light microscope (Olympus, CKX41, Japan). Pictures were taken using an image acquisition system (DP2- SAL; Olympus, Tokyo, Japan).

### 4.5. RNA Isolation and RT-PCR

RNA isolation was performed as previously described [38,39]. Mice were sacrificed 24 h after last challenge and mice ear was used for gene expression analysis. Real-time PCR analysis was performed with duplicate using SYBR^®^ Master Mix in the BIOER Real-Time PCR machine (Fluorescent Quantitative Detection systems; Hangzhou, China). For calculation efficiency of the amplification, the relative quantitative of each target gene was normalized to the housekeeping gene as β-Actin. Data was calculated by the 2^-△△CT^ method based on the normalization gene of control group [40]. Primers used for RT-PCR are listed in Table S2. 

### 4.6. Serum IgE ELISA

Total IgE ELISA was performed as previously described [37]. Serum was collected from the aorta of mice. Whole blood was centrifuged at 4 °C for 15 min at 12,000 rpm. Samples were diluted to 1/200 with PBS prior to ELISA. ELISA kit was purchased from BD Pharmingen (San Diego, CA, USA). All measurements were analyzed by optical density at 450 nm.

### 4.7. Histamine Release Assay

Histamine concentrations in serum were measured using mouse enzyme-linked immunosorbent assay (ELISA) kits according to the manufacturer’s instructions. (Abcam, Cambridge, UK).

### 4.8. Cells and Reagents

NIH3T3/NKκB-luc cell line was purchased from Panomics (RC0015). Cells were maintained in a humidified incubator at 37 °C and 5% CO_2_. Recombinant Human TNF-alpha was purchased from Peprotech (300-01A-10, Peprotech). Bay was purchased from Sigma (11-7082, Sigma). Perphenazine was purchased from TCI (P1970, TCI).

### 4.9. Cell Viability Assay

Viability test was performed as previously described with slight modification [41,42]. Briefly, 1 × 10^4^ cells were plated in a 96-well plate. Eight hours after perphenazine treatment, cells were incubated with a mixture (1:10) of EZ-Cytox cell viability assay kit (Dogen, EZ3000) and Fibroblast Growth Basal Medium (CC-3131, Lonza). Then, the plate was incubated for 30 min in the incubator and determined absorbance at 450 nm with reference to 655 nm wavelength (iMark, Biorad).

### 4.10. Luciferase Assay

Luciferase assay was performed as previously described with slight modification [43]. 1 × 10^4^ cells were seeded in 96-well plates treated 25 ng/mL TNF-α for 8 h with or without perphenazine. Cell extracts were prepared using 60 µL of passive lysis buffer (Promega). Luciferase activities were measured using Veritas Luminometer (Turnur Designs, Sunnyvale, CA, USA).

### 4.11. Immunofluorescence

Immunofluorescence was performed as previously described with slight modification [37]. Slides were incubated in blocking buffer (BLOXALL^®^ Endogenous Peroxidase and Alkaline Phosphatase Blocking Solution, Vector Laboratories, Inc., CA, USA) for 1 h at room temperature to remove non-specific binding. Next, they were incubated for 24 h with dopamine receptor D2 (D2DR) antibody (Santacruz biotechnology, Texas, USA) in blocking buffer at 4 °C.

### 4.12. Web-Based Meta-Analysis

Microarray datasets from studies (GSE120721 [44] and GSE6012 [45] were analyzed using GEO2R (https://www.ncbi.nlm.nih.gov/geo/geo2r) to determine the levels of dopamine receptor expression.

### 4.13. Statistical Analysis

All statistical evaluations were performed using Prism 6 (GraphPad Software, La Jolla, CA, USA). Data are given as mean ± standard error of the mean (SEM). Statistical significance was analyzed using Student’s *t*-test and one-way ANOVA followed by Tukey’s post analysis. *p* values of < 0.05, < 0.01, < 0.005 and < 0.001 were considered as statistically significant differences.

## Supplementary Materials

Supplementary materials can be found at https://www.mdpi.com/1422-0067/21/9/3241/s1.
