# Supplementary material for: Perphenazine Attenuates the Pro-Inflammatory Responses in Mouse Models of Th2-Type Allergic Dermatitis
Source: Int J Mol Sci. 2020 May 3;21(9):3241. doi: 10.3390/ijms21093241 (PMC7247351; doi:10.3390/ijms21093241)
Supplement: Supplementary file 1 [file ijms-21-03241-s001.pdf]

Perphenazine Attenuates the Pro-Inflammatory Responses in Mouse Models of Th2 Type Allergic Dermatitis

Min-Jeong Heo, Soo Young Choi, Chanmi Lee, Yeong Min Choi, In-sook An, Seunghee Bae, Sungkwan An and Jin Hyuk Jung

Table 1. Meta-analysis of D2DR expression in dermatitis patients and control subjects.

Table S1. Meta-analysis of D2DR expression in skin biopsy of dermatitis patients and normal control (GSE6012 and GSE120721)

Gene	Imported Id	Rank	Fold Change	P-Value	Test Expression	Control Expression	Samples	GSE number	PMID
D2DR	216924_s_at	1195	1.8	0.046	357.2	198.1	Skin biopsy- atopic eczema _vs_ healthy control	GSE6012	16918518
D2DR	208215_x_at	7753	1.66	0.0016	357.8	215.5	Dermis from lesional skin of atopic dermatitis patients _vs_ normal healthy skin	GSE120721	25567045

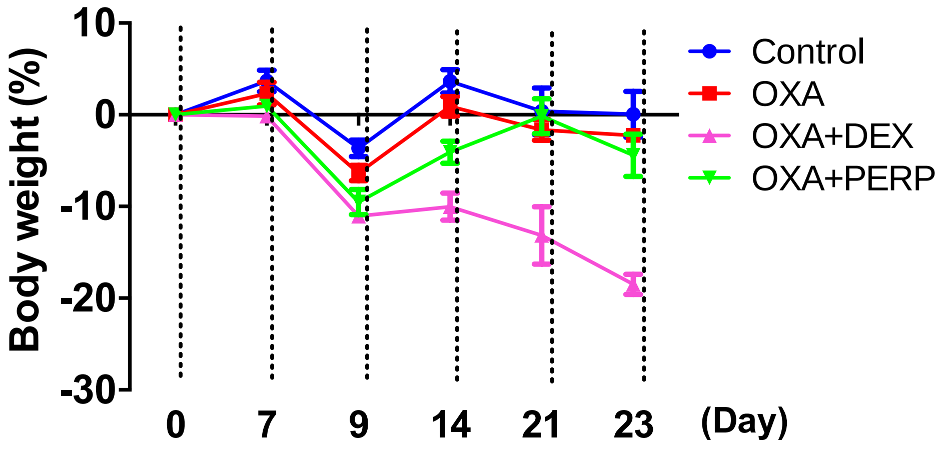

Figure S1. Body weight variation in oxazolone-induced animal model of dermatitis. Mice weight was measured at indicated days and percentage of weight change were calculated based on day 0.

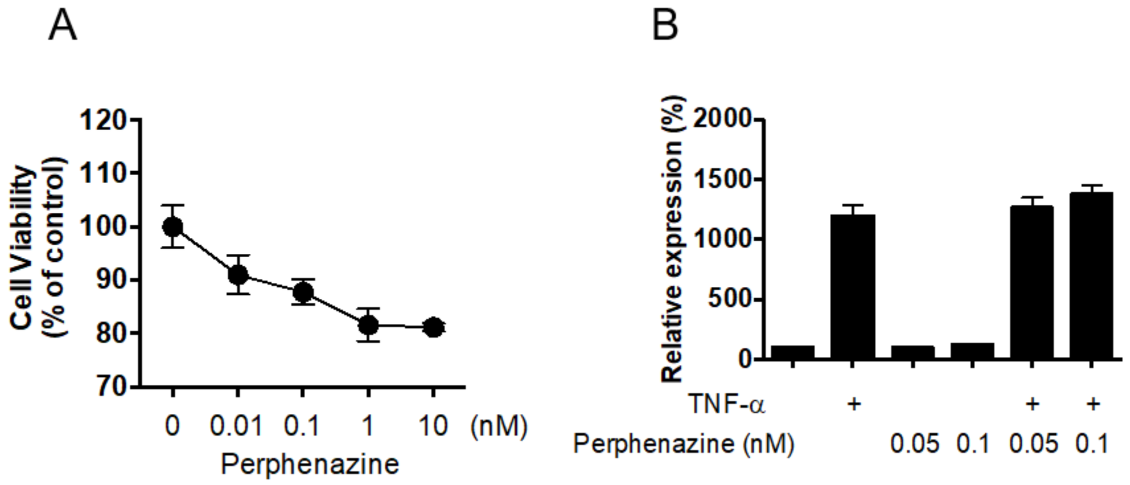

Figure S2. Perphenazine is not able to modulate NFκb activity *in vitro* (**A**) Viability of fibroblasts after indicated concentration of perphenazine treatment for 8h. (**B**) Luciferase assay after 8h of incubation with either TNF-α or TNF-α and perphenazine.

Table S2. List of primers used for Quantitative real-time PCR.

Gene	Forward (5′ to 3′)	Reverse primer (5′ to 3′)
D2DR	TGGCTGCCCTTCTTCATCACGC	TGAAGGCCTTGCGGAACTCAATGT
TNF-α	CTACTCCTCAGAGCCCCCAG	TGACCACTCTCCCTTTGCAG
IFN-γ	CCATCGGCTGACCTAGA	GCCACTTGAGTTAAAATAGTTATTCAGAC
IL-1β	GAAAGACGGCACACCCACCCT	GCTCTGCTTGTGAGGTGCTGATGTA
IL-4	ACAGGAGAAGGGACGCCA	GAAGCCCTACAGACGAGCTCA
IL-5	GGCTGGCCTCAAACTGGTAA	CCCTGATGCAACGAAGAGGA
IL-6	GTGTAATTAAGCCTCCGACTTG	TCCAGTTGCCTTCTTGGGAC
IL-10	ATAACTGCACCCACTTCCCA	GGGCATCACTTCTACCAGGT
IL-13	GCAACGGCAGCATGGTATGGA	TGGTATCGGGGAGGCTGGAGAC
β-Actin	GTATGGAATCCTGTGGCATC	AAGCACTTGCGGTGCACGAT
